# Royal Southern Hospital, Liverpool

**Published:** 1911-01-14

**Authors:** Allen Naldreth

**Affiliations:** Superintendent and Secretary.


### Editor's Letter-Box.

### ROYAL SOUTHERN HOSPITAL, LIVERPOOL.

New Out-patient Department.

*To the Editor of* The Hospital.

Dear Sir,—In answer to the critical description appearing in your issue of the 31st ult., I should like to be allowed to supplement my article with a few remarks.

In giving particulars of requirements to the competing architects the question of a separate patients' exit was not overlooked, but, as good as the principle of guiding patients from entrance to a separate exit may be, I am not alone in thinking that, in a small department such as this, the advantages would be altogether outweighed by the necessity of providing attendants for two doors.

With regard to the scheme of the work, the rooms marked " Consulting Room " are intended for use either for consulting or examination purposes, and will each be provided with a couch—they are made communicating to enable a consultant to sit in the centre room and use the rooms on either side as examination rooms.

It is not intended that a patient using the foot and arm baths shall return to the dressing rooms—dressing boxes are being provided in these rooms, and the patients using them will be dressed there.

As in Liverpool we are well provided with " special " hospitals, the accommodation allotted to special departments is ample, although, as you remark, the one dark room has to do duty for several purposes.

The sanitary offices for patients are screened from direct communication with the corridor by swing doors—by this means and by cross ventilation on the other side of these doors these offices are disconnected from the general air of the department.—Yours faithfully,

Allen Naldreth,

Superintendent and Secretary.

January 4.

